# 20-Day Trend of Serum Potassium Changes in Bam Earthquake Victims with Crush Syndrome; a Cross-sectional Study

**Published:** 2017-01-08

**Authors:** Saeed Safari, Iraj Najafi, Mostafa Hosseini, Alireza Baratloo, Mahmoud Yousefifard, Hamidreza Mohammadi

**Affiliations:** 1 Emergency Department, Shohadaye Tajrish Hospital, Shahid Beheshti University of Medical Sciences, Tehran, Iran.; 2 Department of Nephrology, Dr. Shariati Hospital, Tehran University of Medical Sciences, Tehran, Iran.; 3 Department of Epidemiology and Biostatistics, School of Public Health, Tehran University of Medical Sciences, Tehran, Iran.; 4 Department of Physiology, School of Medicine, Tehran University of Medical Sciences, Tehran, Iran.

**Keywords:** Rhabdomyolysis, crush syndrome, potassium, water-electrolyte imbalance, disaster victims

## Abstract

**Introduction:**

Many of those who survive following an earthquake die in the next phase due to preventable and treatable medical conditions such as hyperkalemia. The present study aimed to evaluate the trend of potassium changes in crush syndrome patients of Bam earthquake.

**Methods:**

In this retrospective cross-sectional study, using the database of Bam earthquake victims, which were developed by Iranian Society of Nephrology following Bam earthquake, Iran, 2003, the 20-day trend of potassium changes in > 15 years old crush syndrome patients was evaluated.

**Results:**

135 crush syndrome patients with the mean age of 29.9 ± 9.91 years were evaluated (56.3% male). Mean potassium concentration during the first 3 days of admission was 5.6 ± 1.3 mEq/L. On the day of admission, 43.1% (95% CI: 34.0 - 52.2) had normal potassium concentration, 3.4% (95% CI: 0.1 - 6.8) had hypokalemia, and 53.4% (44.3 - 62.6) had hyperkalemia. During 20-day follow-up, 62.3% (95% CI: 66.7-71.9) of the patients had normal potassium. While, 11.5% (95% CI: 9.7-13.3) had hypokalemia and 19.2% (95% CI: 17.0-21.5) had hyperkalemia. As the days of hospitalization increased, prevalence of hyperkalemia decreased while hypokalemia increased. On the 17^th^ day 21.2% (95% CI: 2.2-39.9) had hypokalemia and 10.5% (95% CI: 0.1 – 24.7) had hyperkalemia.

**Conclusion:**

Findings of the present study showed that following urine alkalinization and fluid resuscitation, the prevalence of hyperkalemia reduced, but hypokalemia developed. It seems that the correction of serum potassium level should be accompanied by precise monitoring of intake and output of the patient and prescription of reasonable amount of intravenous fluid.

## Introduction

Crush syndrome following traumatic rhabdomyolysis is a life-threatening condition that is accompanied by severe shock, cardiovascular disorders and acute kidney injury. Traumatic rhabdomyolysis is a result of direct pressure on skeletal muscles and is frequently seen in accidents, sports and most importantly in natural disasters such as earthquakes (1-3). Electrolyte abnormalities are among very common problems of these patients. When pressure is removed and perfusion of ischemic body part is restored, intracellular ions are released in the systemic circulation through the damaged cell membrane (4, 5). 

Hyperkalemia is the most important and fatal electrolyte imbalance in these patients (6). Severe hyperkalemia may lead to dysrhythmia, cardiac arrest and finally sudden death. This electrolyte imbalance can occur on the site of accident, during transportation to the hospital and even during hospitalization (7). To control this condition, early and vigorous fluid resuscitation can be helpful and sometime lifesaving (8). However, there are studies that show vigorous fluid resuscitation can leads to a drop in serum potassium level and sometime hypokalemia (9).

Some existing studies have evaluated the changes in the ion concentration of crushed patients in the initial 3 days after earthquake (7, 10, 11). However, there is not enough knowledge regarding long-term changes in the concentration of this ion. Therefore, the present study aimed to evaluate the 20-day trend of potassium concentration changes in crush syndrome patients of Bam earthquake.

## Methods

***Study design and setting***

The present study is a retrospective cross-sectional one designed to evaluate the trend of changes in potassium ion during the 20 initial days of hospitalization in crush syndrome patients of Bam earthquake. This study was approved by the Ethics Committee of Shahid Beheshti University of Medical Sciences, Tehran, Iran. The researchers adhered to the principles of Helisinki Declaration and confidentiality of patient information throughout this study.

***Participants***

To achieve the aims of this study, the data of Bam earthquake victims were used. Following Bam earthquake, Iran, 2003, Iranian Society of Nephrology with the association of International Society of Nephrology, designed a questionnaire and sent it to all the hospitals that were expected to admit the earthquake victims. These hospitals included 15 centers in 7 cities of Bandarabbas, Bushehr, Isfahan, Kerman, Shiraz, Tehran, and Zahedan. Data from Shiraz were not entered to the database since the centers did not cooperate. Data were entered to a database in the same year. The details of data gathering and management have been introduced in previously published studies (1, 12-15).

In the present study, crush syndrome patients whose serum potassium level was recorded for at least the initial 3 days of admission were included. Patients under 15 years old, those with chronic kidney diseases and patients whose creatine phosphokinase (CPK) was never measured were excluded. 

Crush syndrome was defined as traumatic rhabdomyolysis leading to serum creatinine over 1.66 mg/dl and CPK higher than 1000 IU/L in 2 measurements during hospitalization (16). Hypokalemia was defined as serum potassium level lower than 3.5 mEq/L and hyperkalemia as serum potassium level higher than 5 mEq/L (17). Serum potassium levels were evaluated daily and the data was recorded in the database.

***Statistical analysis***

Considering 15.9% prevalence of hyperkalemia (10), 95% confidence interval (CI), and 0.06 accuracy, the sample size was calculated to be 134 cases. Data from the database were entered to STATA 11.0 and analyzed. Serum potassium level was reported as mean ± standard deviation (SD). Patients were classified as normal, hypokalemia and hyperkalemia based on their serum potassium level and the prevalence was reported with 95% CI.

## Results

**Figure 1:**
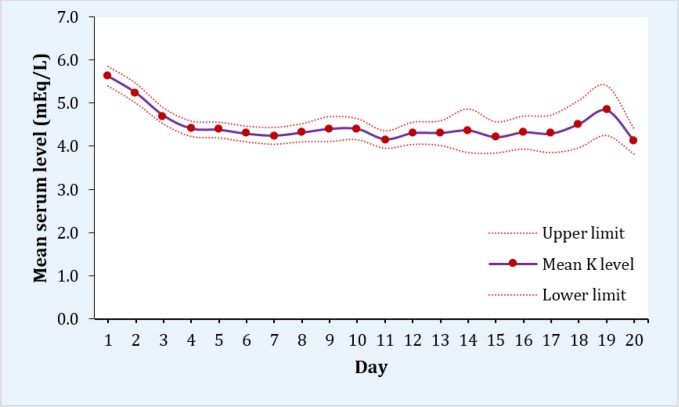
Mean and 95% confidence interval of potassium levels during first 20 days after crush injury.

135 crush syndrome patients with the mean age of 29.9 ± 9.91 years were evaluated (56.3% male). Demographic, clinical, and laboratory findings of the patients are presented in table 1. Mean and SD of potassium concentration during the first 3 days of admission was 5.6 ± 1.3 mEq/L. Trend of changes of mean potassium level with 95% CI showed little variation during the first 20 days of admission (figure 1). On the day of admission, 43.1% (95% CI: 34.0 - 52.2) had normal potassium concentration, 3.4% (95% CI: 0.1 - 6.8) had hypokalemia, and 53.4% (44.3 - 62.6) had hyperkalemia. During 20-day follow-up, 62.3% (95% CI: 66.7-71.9) of the patients had normal potassium. While, 11.5% (95% CI: 9.7-13.3) had hypokalemia and 19.2% (95% CI: 17.0-21.5) had hyperkalemia.

**Figure 2:**
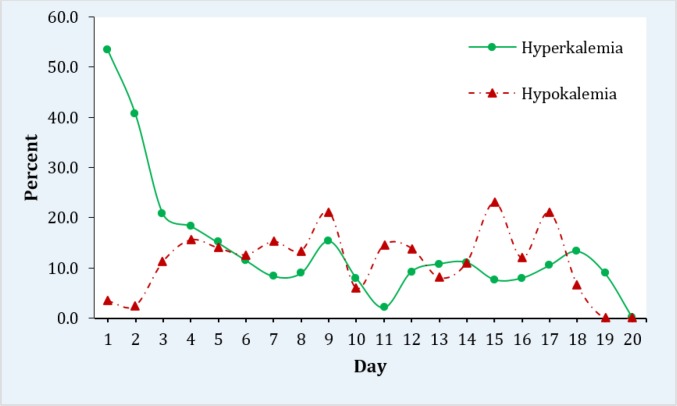
Trend of hyperkalemia and hypokalemia prevalence among studied patients

As the days of hospitalization increased, prevalence of hyperkalemia decreased while hypokalemia increased. On the 17^th^ day 21.2% (95% CI: 2.2-39.9) had hypokalemia and 10.5% (95% CI: 0.1 – 24.7) had hyperkalemia. At the end of the 20^th^ day, all cases of hypo and hyperkalemia were corrected (table 2 and figure 2).

**Table 1:** Baseline characteristics of included patients

**Variable**	**Values**
**Age (year)**	
15-34	105 (79.2)
35-54	17 (12.6)
≥55	11 (8.2)
**Gender **	
Male	76 (56.3)
Female	59 (43.7)
**Time under the rubble (hours)**	6.2 ± 3.4
**Systolic blood pressure** ** (mmHg)**	126.8 ± 2.6
**Diastolic blood pressure** ** (mmHg)**	77.9 ± 1.0
**Fluid intake (ml)**	2872.0 ± 1829.0
**Urine output (ml)**	897.0 ± 923.0
**Blood urea nitrogen (mg/dL)**	104.0 ± 59.0
**Creatinine** **(mg/dL)**	4.73 ± 2.3
**Creatine phosphokinase** **(IU/L)**	17.4 ± 24.7
**Lactate dehydrogenase** **(U/L)**	3.1 ± 2.5
**Uric acid** **(mg/dL)**	8.5 ± 2.8

Data were presented as mean ± standard deviation or number (%).

**Table 2:** Prevalence of hypokalemia, normal, and hyperkalemia among Bam earthquake victims during 20 days of admission

**Day**	**Normal**		**Hypokalemia**			**Hyperkalemia**	
	Prevalence	95% CI	Prevalence		95% CI	Prevalence	95% CI
1	43.1	34.0 - 52.2	3.4	0.1 - 6.8		53.4	44.3 - 62.6
2	56.8	47.8 - 65.8	2.5	0.0 - 5.4		40.7	31.8 - 49.6
3	67.9	59.0 - 76.9	11.3	5.3 - 17.4		20.8	13.0 - 28.5
4	66.1	57.1 - 75.0	15.6	8.7 - 22.4		18.3	11.0 - 25.7
5	71.0	62.1 - 79.9	14.0	7.2 - 20.8		15.0	8.0 - 22.0
6	75.9	66.8 - 84.9	12.6	5.6 - 19.7		11.5	4.7 - 18.2
7	76.4	66.5 - 86.3	15.3	6.9 - 23.7		8.3	1.9 - 14.8
8	77.6	67.5 - 87.7	13.4	5.2 - 21.7		9.0	2.1 - 15.9
9	63.5	50.2 - 76.7	21.2	9.9 - 32.4		15.4	5.5 - 25.3
10	86.0	76.3 - 95.7	6.0	0.0 - 12.7		8.0	0.1 - 15.6
11	83.3	72.7 - 94.0	14.6	4.5 - 24.7		2.1	0.1 - 6.2
12	76.7	64.0 - 89.5	14.0	3.5 - 24.4		9.3	0.5 - 18.1
13	81.1	68.3 - 93.9	8.1	0.1 - 17.0		10.8	0.7 - 21.0
14	77.8	61.8 - 93.8	11.1	0.1 - 23.2		11.1	0.1 - 23.2
15	69.2	51.1 - 87.3	23.1	6.5 - 39.6		7.7	0.1 - 18.1
16	80.0	64.0 - 96.0	12.0	0.1 - 25.0		8.0	0.1 - 18.9
17	68.4	46.9 - 89.9	21.1	2.2 - 39.9		10.5	0.1 - 24.7
18	80.0	70.0 - 90.0	6.7	0.1 - 19.7		13.3	0.1 - 25.1
19	91.0	88.0 - 96.0	0.0	0.1 - 0.0		9.0	0.6 - 16.0
20	100.0	90.0 - 100.0	0.0	0.0 - 0.0		0.0	0.0 - 0.0
**Total**	**69.3**	**66.7 – 71.9**	**11.5**	**9.7-13.3**		**19.2**	**17.0-21.5**

CI: Confidence interval.

## Discussion

Findings of the present study showed the 53.4% prevalence of hyperkalemia and 3.4% hypokalemia in crush syndrome patients of Bam earthquake victims on the day of admission. Following therapeutic interventions, urine alkalinization and fluid resuscitation, the prevalence of hyperkalemia decreased but hypokalemia increased. 

Clinical and laboratory variables monitoring in the earthquake victims can be helpful in rapid diagnosis and efficient management of these patients (18-22). The frequent cause of mortality at the scene of disaster is direct trauma. However, many of those who survive die in the next phase due to preventable and treatable medical conditions such as hyperkalemia. Hyperkalemia is the most important electrolyte that can lead to a higher rate of mortality by causing fatal arrhythmias (7, 10, 11, 23, 24). 

A study by Sever et al. on crush syndrome patients after Marmara earthquake in Turkey showed that hyperkalemia is the most important life-threatening problem in patients with traumatic crush syndrome (25). Studying the causes of mortality in crush syndrome patients in Marmara earthquake in Turkey showed that 42% of the cases that died due to crush syndrome had hyperkalemia (7). Oda et al. analyzed data of those who were injured in Hanshin-Awaji earthquake in Japan and introduced hyperkalemia as one of the most important causes of death among patients with crush syndrome (11). However, data of Wenchuan earthquake in China show that hypokalemia (18.2%) was more common than hyperkalemia (15.9%). Researchers believe that this has happened since most of those with hyperkalemia have died on the way to hospital as there was not enough fluid for early fluid resuscitation on the scene in Wenchuan earthquake (10). 

However, hypokalemia is a problem that has not been studied properly. Early and vigorous fluid resuscitation can prevent acute kidney injury in earthquake victims with crush syndrome. In addition, it can reduce the severity of hyperkalemia and prevent it from leading to fatal arrhythmias (9). For instance, Gunal et al. studied 15 victims of Bingol earthquake and showed that on admission, 9 had hypokalemia, while only one had hyperkalemia. These researchers believe this to be due to fluid resuscitation and bicarbonate prescription on the scene (9). In Bam earthquake, lack of enough fluid for fluid resuscitation on the scene led to lack of fluid resuscitation during the initial hours of the incident for many of the victims. Therefore, hyperkalemia was very common on the first day. However, during the course of hospitalization fluid resuscitation reduced the prevalence of hyperkalemia. Yet, too much fluid resuscitation led to hypokalemia. Although all cases of hypo and hyperkalemia were resolved on the 20^th^ day, since hypokalemia may be accompanied by the probability of cardiotoxicity, precise monitoring of intake and output of the patient and prescription of reasonable amounts of intravenous fluid can be important in correcting this imbalance and preventing it. Excessive fluid resuscitation during hospitalization can be prevented by adjusting fluid resuscitation based on laboratory findings and serum ion levels of the patients.

## Limitation:

Missing data, outdated data, referring the victims to different health centers, different therapeutic and resuscitative approaches, variations in management of the patient in these centers, and not exactly knowing the type of trauma and its severity are among the most important limitations of this study. 

Despite all these limitations, since our information regarding management and outcome of patients with crush syndrome is limited, the authors decided to study and publish the minimum existing data.

## Conclusion:

Findings of the present study showed that following urine alkalinization and fluid resuscitation, the prevalence of hyperkalemia reduced, but hypokalemia developed. It seems that correction of serum potassium level should be accompanied by precise monitoring of intake and output of the patient and prescription of reasonable amount of intravenous fluid.
